# An Atypical Zenker’s Diverticulum: A Case Report

**DOI:** 10.7759/cureus.75595

**Published:** 2024-12-12

**Authors:** Abdoulaye Maman Bachir, Salissou Iro, Adakal Ousseini, Hamissou Souley Maman Noury, Issa Abdoulhaziz

**Affiliations:** 1 Surgery Department, Dan Dicko Dankoulodo University of Maradi, Maradi, NER; 2 General Surgery Department, Niger Maradi Reference Hospital, Maradi, NER; 3 Otolaryngology - Head and Neck Surgery, General Hospital of Maradi, Maradi, NER; 4 Surgery Department, Dan Dicko Dankoulodo University of Maradi, Faculty of Health Sciences, Maradi, NER; 5 Anesthesiology and Critical Care and Emergency Department, Niger Maradi Reference Hospital, Maradi, NER; 6 Medical Speciality Department, Niger Maradi Reference Hospital, Maradi, NER

**Keywords:** esophagus, externalized, multidisciplinary, surgery, zenker’s diverticulum

## Abstract

A Zenker's diverticulum (ZD) is an acquired hernia of the mucosa and submucosa at the pharyngoesophageal junction dorsally through Killian's triangle, considered a zone of weakness. The authors report their experience in the management of a case of a ZD with oral externalization following coughing. Surgery made by a multidisciplinary team consisted first of resection of the edematous exteriorized portion of the diverticulum. Second, endoscopic assistance helped to reduce the remaining portion of the diverticulum, and a right anterolateral cervicotomy allowed to remove it at the origin. Immediate and medium-term postoperative follow-ups were uneventful. Functional and endoscopic results were satisfactory. A ZD is a rare condition that can take confusing or even enigmatic forms, making it a real challenge in terms of management.

## Introduction

A Zenker's diverticulum (ZD) is an acquired protrusion of the mucosa and submucosa located dorsally at the pharyngoesophageal junction at an area of muscular weakness: Killian's triangle [1]. The reported prevalence ranges from 0.01% to 0.11%. It generally occurs in patients between middle age and the elderly [2]. The most predominant symptoms are dysphagia and regurgitation [3]. Treatment is recommended for symptomatic patients, and considering the etiopathogenesis of the disease, myotomy of the cricopharyngeal muscle has been the conventional treatment for a long time, with satisfactory results. Myotomy can be performed using open or endoscopic surgical approaches. However, this is associated with high complication rates. Endoscopic repair of a ZD has emerged recently as a viable, safe, and effective option [4,5].

Endoscopic stapled diverticulotomy is the preferred approach, but flexible endoscopy is an attractive option, particularly for high-risk patients [4,5].

Here, we report the case of a ZD with an atypical clinical presentation managed at the Maradi Reference Hospital.

## Case presentation

**Figure 1:**
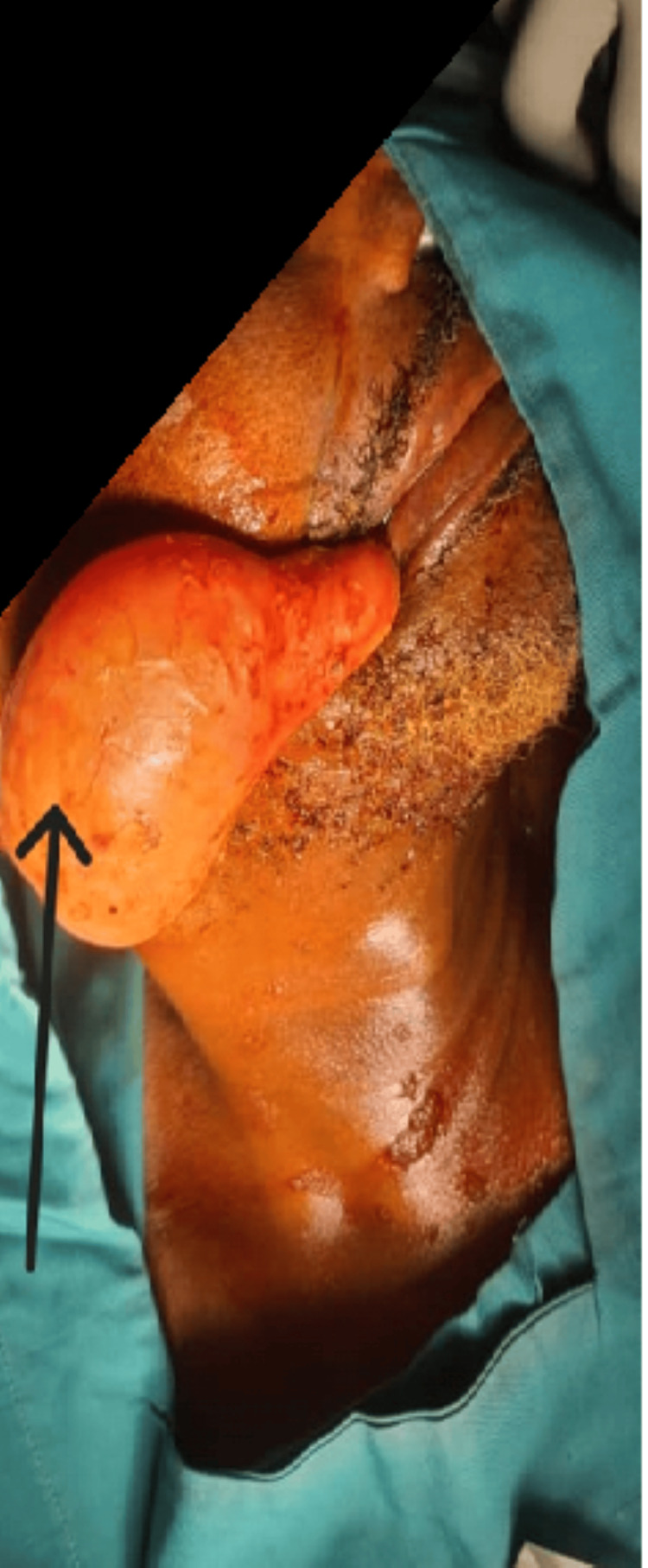
Zenker’s diverticulum exteriorized (black arrow).

A 70-year-old patient was seen as an outpatient in stomatology for a pale pink, saccular tissue mass, whose surface macroscopically mimics the oropharyngeal epithelium. This mass was prolapsed and externalized through the buccal orifice (Figure 1).

No ventilatory or hemodynamic disturbances were checked. The externalized part of the mass was edematous and measured 15 cm in length by 7 cm in greatest width. The patient reported an acute episode of dry cough a week ago. The mass was externalized following a coughing effort. The patient complains of permanent halitosis since a year ago. The patient was presented for upper gastrointestinal endoscopy, which easily revealed the connection of the mass to the area of pharyngoesophageal weakness. The diagnosis of an externalized ZD was therefore retained, and the patient was prepared and admitted to the operating theater by a multidisciplinary team: general and visceral surgeon, ENT, maxillofacial surgeon, gastroenterologist, endoscopist, and anesthesiologist.

The patient lay supine under general anesthesia with nasotracheal intubation, neck in extension, and head in left lateral rotation.

We proceeded with disinfection and sterile draping, followed by a right pre-sternocleidomastoid incision and then dissection of the cellulomembranous spaces and exposure of the cervical esophagus in its initial portion. First, the prolapsed part of the ZD, which was highly oedematous and impossible to reduce, was ligated and resected.

**Figure 2:**
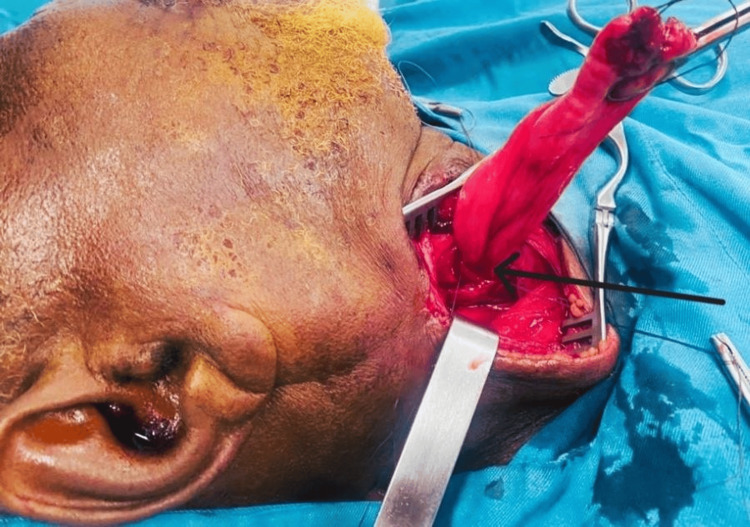
Reduction of the remaining part of the Zenker’s diverticulum. Black arrow at the junction with the esophagus.

Then, using an endoscopic approach, the residual stump was reduced and exteriorized through the cervical incision (Figure 2), and a nasogastric tube was placed under endoscopic control.

**Figure 3:**
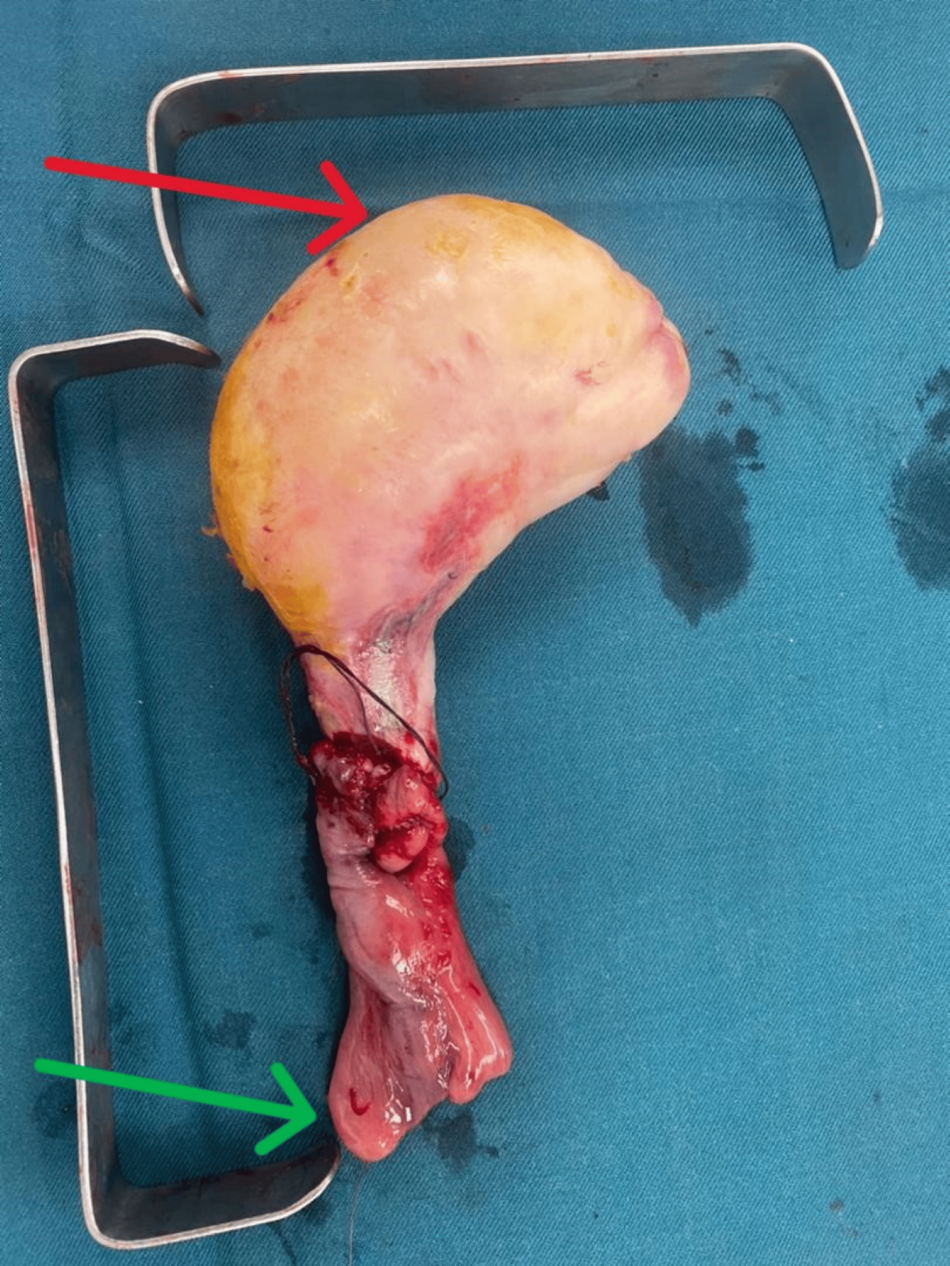
View of the surgical specimen. Red arrow = free edge. Green arrow = basis.

The base of the remaining ZD was dissected, the diverticulectomy was completed (Figure 3), and the esophageal wall sutured in two planes.

**Figure 4:**
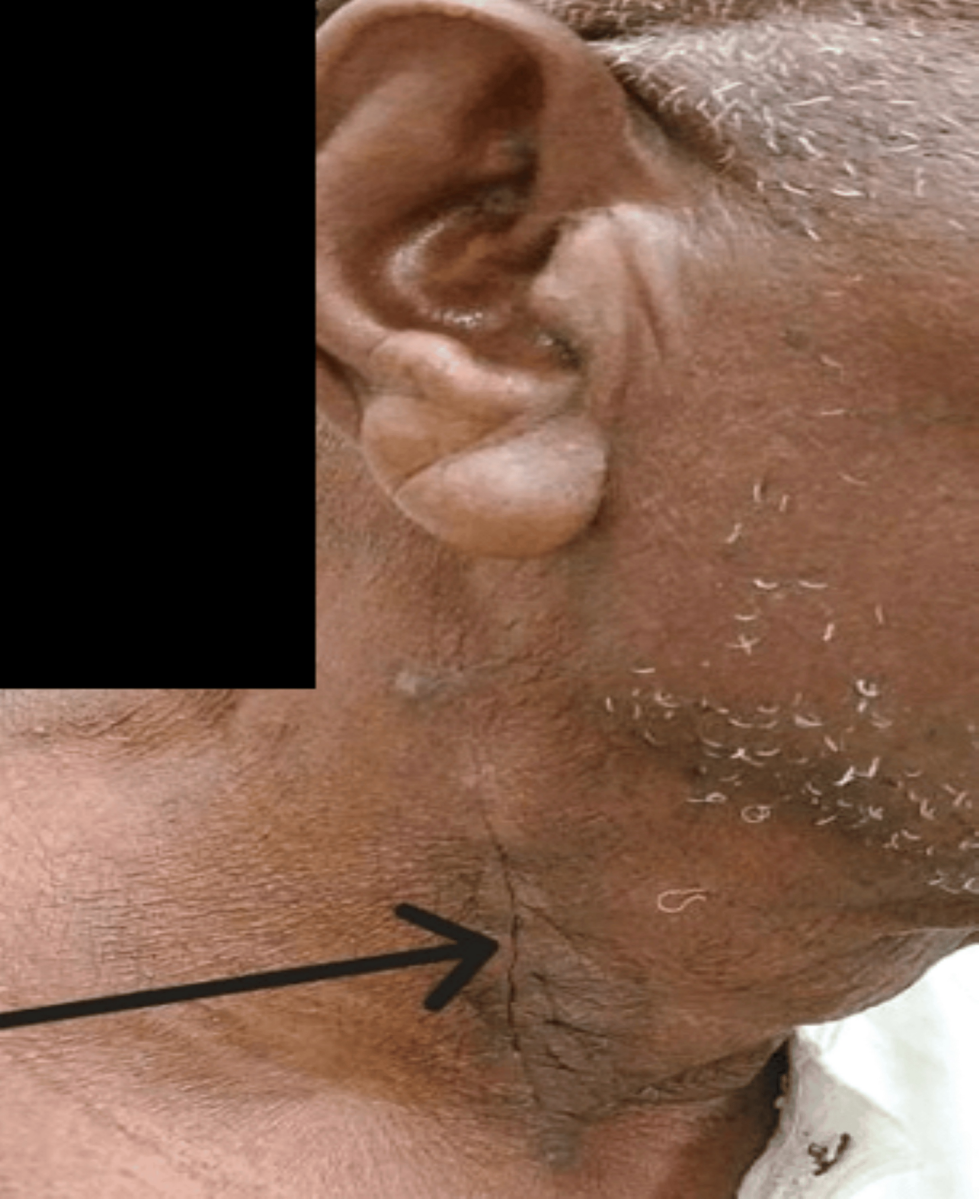
Patient seen after two weeks of follow-up. Black arrow at the surgical scar.

Next, we sectioned the fibers of the cricopharyngeal muscle. Plane-by-plane closure is performed on a suction drain. The nasogastric tube was left in place for five days, and the patient was fed via this tube. Postoperative management was simple. The patient was discharged home seven days after surgery. A clinical check-up was performed at 15 and 30 days (Figure 4), followed by an endoscopic check-up after six weeks.

Histological examination revealed esophageal-type mucosa with fibrous chorion and lymphoplasmacytic infiltrate, with no evidence of malignancy.

## Discussion

Typically, ZD is a condition with clearly defined management guidelines. Nevertheless, there are various clinical forms, including oral externalization, which occurs during coughing stress, as in the present case. This is a "glove finger-like" eversion of a classic ZD due to a lack or weakness in fibrous elements binding the mucosal and submucosal hernial sac with the surrounding cellulo-membranous tissue, under the effect of high pressure produced at the pharyngo-oesophageal carrefour during coughing [6].

According to the literature, it is a pathology of the elderly, appearing between the seventh and eighth decades of life, and exceptionally before the fourth decade [7]. ZD can therefore be seen in black subjects but was classically described in the White race and more often in females [8]. Thus, three main elements seem to clearly emerge in the pathogenesis of the diverticulum. These are anatomical considerations linked to the zone of weakness [9], morphofunctional disorders due to pharyngoesophageal neuromuscular dysfunction [10], and pharyngosphincteric asynergy secondary to neurological damage, which seems to explain the occurrence of ZDs in elderly subjects [11].

In our case, the patient was asymptomatic before externalization of the diverticulum. In fact, 15-20% of them are discovered by chance during a barium transit [12]. The diagnosis in this context of the externalization of this mass was suspected clinically and confirmed by upper endoscopy. The patient consulted in a context of "social emergency" due to the appearance of an atypical mass never seen elsewhere in his immediate environment, running the risk of appearing as an alien, with the attendant risk of stigmatization. The treatment for this relative emergency was surgical, as edema had set in, disallowing any reduction trial using an endoscopic approach. This treatment was carried out in two stages, oral to reduce the volume of the ZD, then cervical for the definitive treatment, which consisted of a hybrid procedure: endoscopic for reduction of the remaining end of the ZD using a landmark suture left on the section margin of the stump through the cervical approach in the zone of weakness at the pharyngoesophageal junction, combined with right lateral esophagotomy. This enabled complete dissection of the stump, resection and suturing of the esophageal wall in two planes, and myotomy of the cricopharyngeal according to the technique described [13]. No complications were reported postoperatively with a three-month follow-up.

## Conclusions

A ZD is a benign condition and its management is currently well-codified. However, some presentations, such as oral externalization, really challenge the practitioner's experience and call for a pragmatic, multidisciplinary approach. These conditions are the key to satisfactory treatment results. If left untreated, externalized ZD can constitute a situation at risk of degradation and stigmatization, leading to social rejection and withdrawal.
